# The computational power of astrocyte mediated synaptic plasticity

**DOI:** 10.3389/fncom.2012.00093

**Published:** 2012-11-01

**Authors:** Rogier Min, Mirko Santello, Thomas Nevian

**Affiliations:** Department of Physiology, University of BerneBerne, Switzerland

**Keywords:** astrocytes, synaptic plasticity, spike-timing-dependent plasticity, STDP, metaplasticity, heterosynaptic plasticity, computation, calcium

## Abstract

Research in the last two decades has made clear that astrocytes play a crucial role in the brain beyond their functions in energy metabolism and homeostasis. Many studies have shown that astrocytes can dynamically modulate neuronal excitability and synaptic plasticity, and might participate in higher brain functions like learning and memory. With the plethora of astrocyte mediated signaling processes described in the literature today, the current challenge is to identify, which of these processes happen under what physiological condition, and how this shapes information processing and, ultimately, behavior. To answer these questions will require a combination of advanced physiological, genetical, and behavioral experiments. Additionally, mathematical modeling will prove crucial for testing predictions on the possible functions of astrocytes in neuronal networks, and to generate novel ideas as to how astrocytes can contribute to the complexity of the brain. Here, we aim to provide an outline of how astrocytes can interact with neurons. We do this by reviewing recent experimental literature on astrocyte-neuron interactions, discussing the dynamic effects of astrocytes on neuronal excitability and short- and long-term synaptic plasticity. Finally, we will outline the potential computational functions that astrocyte-neuron interactions can serve in the brain. We will discuss how astrocytes could govern metaplasticity in the brain, how they might organize the clustering of synaptic inputs, and how they could function as memory elements for neuronal activity. We conclude that astrocytes can enhance the computational power of neuronal networks in previously unexpected ways.

Astrocytes represent the largest cellular population in the human brain. It has even been suggested that the increased complexity of human astrocytes strongly adds to the computational power of the human brain (Oberheim et al., 2006). However, the precise function of astrocytes in brain signaling is only partially understood. Astrocytes play a crucial role in brain homeostasis (for review see Simard and Nedergaard, 2004). For example, they take up and redistribute excess extracellular potassium (K^+^) that is released upon high neuronal activity (Chever et al., 2010; Bay and Butt, 2012), and they maintain the brain water balance by taking up or releasing water through water channels (Haj-Yasein et al., 2012). However, in addition to these vital homeostatic functions, recent studies have shown that astrocytes actually engage in active bidirectional interactions with neurons. Research in rodents in the last two decades has highlighted numerous ways in which astrocytes can interact with neurons. In this review, we aim to give an overview of experimental studies on astrocyte-neuron interactions. We focus on the role that astrocytes play in different forms of synaptic plasticity in the healthy brain.

## How are astrocytes activated?

**Figure 1:**
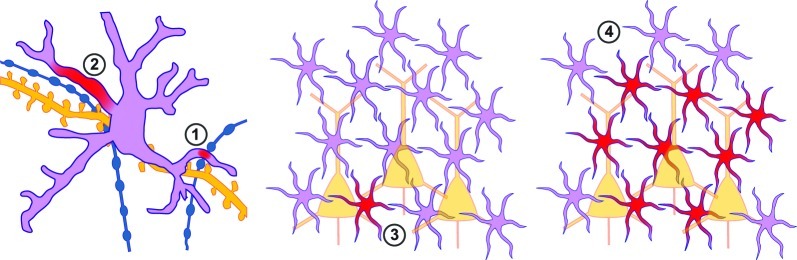
**Spatial properties of astrocytic Ca^2+^ signals. Left:** close-up view of a single astrocyte (pink) together with presynaptic (blue) and postsynaptic (yellow) neuronal elements. (1) Local Ca^2+^ signals (spread ~4 μm) can occur upon activity at a putative single neighboring synapse. (2) More robust regional events (spread ~12 μm) occur presumably when an astrocyte process senses release at several neighboring sites simultaneously. **Middle, Right:** zoomed out view of a network of astrocytes (pink) and neurons (yellow). Upon stronger stimulation an astrocyte Ca^2+^ signal can be generalized to the entire astrocyte (3), or spread through a network of neighboring astrocytes (4).

Astrocytes are strategically positioned to sense ongoing brain activity, i.e., a single astrocyte tightly ensheathes tens of thousands of synapses, hundreds of axons and dendrites and several neuronal somata (Bushong et al., 2002; Halassa et al., 2007). The fine processes of astrocytes make tight associations with pre- and post synaptic neuronal elements, thereby forming the so-called tripartite synapse (Araque et al., 1999). Because astrocytes are electrically passive, their signaling is fundamentally different from neuronal signaling. Instead of integrating membrane depolarization and hyperpolarization into action potential output, like neurons do, astrocytes sense and integrate information mainly through the generation of intracellular calcium (Ca^2+^) signals (Figure 1). It is now well-established that astrocytes are able to sense transmitters released by neurons and other glial cells (either astrocytes or microglia), displaying a wide variety of intracellular responses ultimately leading to a physiological feed-back. Indeed, receptors for all major neurotransmitters seem to be present on the astrocytic plasma membrane *in situ* (Volterra and Meldolesi, 2005), and they can be activated during brain activity *in vivo* (Wang et al., 2006). Activation of these receptors leads to an intracellular Ca^2+^ increase and subsequent release of transmitters from the glial cell (called gliotransmitters), a feedback that is believed to be important for several forms of synaptic plasticity and memory storage (Perea et al., 2009).

The main classes of transmitter receptors that can be found on astrocytic processes are G-protein coupled receptors (GPCRs). Their activation leads to the intracellular activation of phospholipase C (PLC), followed by the production of the messenger molecule inositol trisphosphate (IP_3_) which subsequently induces release of Ca^2+^ from the astrocyte endoplasmic reticulum (ER). Thanks to those receptors astrocytes are not only capable to respond to the classical neurotransmitters glutamate and γ-aminobutyric acid (GABA) released from synapses [through metabotropic glutamate receptors (mGluRs) mGluR1-5 and GABA_B_ receptors respectively; Pasti et al., 1997; Kang et al., 1998] but also to neuromodulators like acetylcholine (Takata et al., 2011; Navarrete et al., 2012) and dopamine (Khan et al., 2001). Moreover, purines like adenosine-5′-triphosphate (ATP) and adenosine (co-)released from synapses or other astrocytes can be sensed by astrocytes (Jourdain et al., 2007; Santello et al., 2011) and play a role in the propagation/amplification of Ca^2+^ signals through the astrocytic syncytium (Poskanzer and Yuste, 2011) or serve as a signal for microglial reaction (Haynes et al., 2006). In addition to sensing axonal release of transmitters, astrocytes have been shown to respond to so-called retrograde signals: transmitters of postsynaptic or dendritic origin (Bernardinelli et al., 2011). For example, strong evidence exists for astrocytes being stimulated by endocannabinoids acting on astrocytic cannabinoid CB_1_ receptors (CB_1_Rs; Navarrete and Araque, 2008, 2010; Min and Nevian, 2012).

In addition to GPCRs, other pathways have been shown to lead to astrocyte Ca^2+^ signals. Astrocytes can possess ligand-gated ion channels whose activation can lead to direct Ca^2+^ influx. For instance, specialized cerebellar astrocytes called Bergmann glia possess Ca^2+^ permeable AMPA type glutamate receptors (AMPARs; Burnashev et al., 1992) which sense ectopic release of glutamate from glutamatergic fibers (Matsui et al., 2005). Other channels that have been shown to mediate astrocyte signals are purinergic P2X channels (Lalo et al., 2008) and transient receptor potential (TRP) channels (TRPC) (Malarkey et al., 2008), and TRPA (Shigetomi et al., 2011). Finally, GABA and glutamate transporter activity linked to Na^+^/Ca^2+^ exchangers (Rojas et al., 2007; Doengi et al., 2009) as well as connexin hemichannel opening (Torres et al., 2012) have been shown to play a role in astrocytic Ca^2+^ signaling.

It should be noted that Ca^2+^ signaling is not the only form of “excitability” displayed by astrocytes. For example, astrocytes also show dynamic sodium (Na^+^) transients mediated by channels, exchangers, and transporters (for recent review see Kirischuk et al., 2012). An important source of activity dependent Na^+^ influx into astrocytes is the Na^+^ that is cotransported by the astrocytic glutamate transporters when astrocytes clear away glutamate from the extracellular space (Chatton et al., 2000).

## The spatiotemporal characteristics of astrocytic Ca^2+^ signals

When stimulated with specific metabotropic receptor agonists, astrocytes display prominent and extremely slow (up to 10 s of seconds) whole-cell Ca^2+^ responses. This is also true for *in vivo* experiments, where sensory stimulation reliably induces astroglial slow Ca^2+^ transients (Wang et al., 2006) sometimes related to vascular responses (Petzold et al., 2008). The recorded Ca^2+^ signal can remain restricted to a single or few astrocytes responding to specific sensory stimuli (Wang et al., 2006; Schummers et al., 2008). Additionally, since astrocytes form complex networks through gap-junctional coupling with neighboring astrocytes (for review see Giaume, 2010; Giaume et al., 2010) Ca^2+^ signals can spread like a wave through the astrocyte network (Nimmerjahn et al., 2009; Kuga et al., 2011). Although the mechanisms underlying the propagation of such Ca^2+^ waves are not fully understood, transport of either IP_3_ or Ca^2+^ itself through gap-junctions may play an important role (Venance et al., 1997). Furthermore, regenerative activity through astrocytic release of signaling molecules like ATP, which in turn activate Ca^2+^ signals in neighboring astrocytes, can be involved in Ca^2+^ wave propagation (Guthrie et al., 1999).

The temporal characteristics of astrocytic Ca^2+^ transients have led to the idea that unlike neurons, astrocytes display exclusively particularly slow responses, and that their signals are not suited to be restricted to small cellular compartments, as happens for example, in dendritic spines. However, *in vivo* experiments have shown that faster local responses of astrocytes in the somatosensory cortex can occur upon hind limb stimulation (time scale of hundreds of ms; Winship et al., 2007). Recently, localized Ca^2+^ activity has been thoroughly studied *in vitro*. Two parallel studies have indeed identified small and relatively fast Ca^2+^ signals that are restricted to the astrocyte process (Di Castro et al., 2011; Panatier et al., 2011). Two main classes of local calcium events have been identified: focal highly confined transients (about 4 μm) and more robust regional events (about 12 μm; Figure 1; Di Castro et al., 2011). The more local events have been proposed to be generated by spontaneous single vesicle release at individual synapses whereas the expanded events seem to be generated by single action potentials activating several neighboring synapses in the astrocyte domain. Remarkably, only the latter have been proposed to have an influence on synaptic transmission, giving, for the first time, some clues to decipher the language the astrocyte employs to communicate with synapses (Di Castro et al., 2011; Panatier et al., 2011).

Another recent study has described an additional form of localized Ca^2+^ events in hippocampal astrocytes. These so-called spotty Ca^2+^ signals spread with a half width of about 5 μm, and last for several seconds (Shigetomi et al., 2011). Unlike most other described astrocyte Ca^2+^ signals, spotty Ca^2+^ signals are mediated by spontaneous openings of a Ca^2+^ permeable channel on the astrocyte membrane, the transient receptor potential A1 (TRPA1) channel. TRPA1 mediated spotty Ca^2+^ signals seem to play an important role in setting the basal intracellular Ca^2+^ concentration of hippocampal astrocytes (Shigetomi et al., 2011).

Because astrocytic Ca^2+^ signals can be very localized, this raises the question whether the distribution of receptors over the astrocyte membrane is homogeneous, or whether astrocytes show hot-spots for certain receptors. This question remains largely unanswered so far, but some interesting results have recently been obtained. For example, hippocampal astrocytes can be activated by agonists of both PAR-1 as well as P2Y_1_ receptors. Both agonists induce somatic Ca^2+^ transients in the astrocyte which look similar, albeit with a different time-course. But surprisingly only astrocytic PAR-1 receptor activation, and not P2Y_1_ receptor activation, leads to astrocyte mediated slow inward currents through NMDA receptors in neighboring neurons (Shigetomi et al., 2008). This suggests that the coupling of these two receptors to intracellular signaling cascades is different, and argues for subcellular differentiation of receptors in astrocytes. Furthermore, a recent study on the distribution of mGluRs on the astrocyte membrane shows that mGluRs show an increased density on astrocyte processes as compared to the soma (Arizono et al., 2012), while another recent study used immunocytochemistry and glutamate uncaging to show that metabotropic glutamate receptors cluster together at discrete locations along astrocytic processes (Panatier et al., 2011).

As mentioned earlier, astrocytic Ca^2+^ elevations can be extended and generalized (involving one entire astrocyte or spreading through several of them; Figure 1). In this way, activity in one astrocyte could spread through a network of neighboring astrocytes. However, either trains of sustained stimulation of synaptic activity (Grosche et al., 1999; Matyash et al., 2001) or a large number of activated fibers (Honsek et al., 2012) are necessary to induce this type of astrocytic Ca^2+^ activity. *In vivo* it has been suggested that astrocytes can synchronize their activity in clusters of 2–5 astrocytes (Hirase et al., 2004; Sasaki et al., 2011a) or spread through a network consisting of dozens to hundreds of astrocytes (Hoogland et al., 2009; Nimmerjahn et al., 2009; Kuga et al., 2011).

Understanding Ca^2+^ signaling in astrocytes has very much been aided by the development of mathematical models. Such models offer a good description of intrinsic and neurotransmitter evoked Ca^2+^ dynamics in astrocytes (De Pittà et al., 2009), as well as the contribution of both gap-junctions (Venance et al., 1997; Kazantsev, 2009; Goldberg et al., 2010; Matrosov and Kazantsev, 2011) and intercellular ATP signaling (Macdonald et al., 2008) to the spread of Ca^2+^ waves through the astrocyte network. However, the experimental data upon which many of these models are based often stems from studies on cultured astrocytes, which have a very simplified morphology compared to the *in vivo* situation. Furthermore, the majority of Ca^2+^ imaging studies performed to date have focused on Ca^2+^ dynamics in the astrocyte soma. As described in the previous paragraphs, high-resolution Ca^2+^ imaging data obtained recently has shown the complicated subcellular interplay between synaptic activity and localized astrocytic Ca^2+^ signals (Di Castro et al., 2011; Panatier et al., 2011). A challenge for the future will be to use a combination of high-resolution imaging and mathematical modeling to understand how such localized signals arise, and how they relate to the more global Ca^2+^ signals occurring in astrocyte networks.

## How do astrocytes signal back to neurons?

As discussed in the previous section, astrocytes can sense a wide variety of neurotransmitters and signaling molecules, and respond with increased Ca^2+^ signaling. But how do astrocytes signal back to neurons? Broadly speaking, astrocytes can do this through three separate mechanisms. Firstly, because astrocytes are crucial for ion homeostasis, they can influence neurons by dynamically altering the ionic balance. Secondly, astrocytes can alter neuronal functioning by modulating the uptake of neurotransmitter molecules from the extracellular space (Theodosis et al., 2008). Thirdly, astrocytes can release transmitters themselves (Araque et al., 2001). We will briefly describe these three mechanisms.

As mentioned earlier, astrocytes are crucial for the passive homeostatic regulation of extracellular ions like potassium. This astrocytic function is essential for brain homeostasis. Furthermore, if astrocytes would actively modulate the uptake of extracellular ions this would endow them with a powerful way to modulate neuronal excitability. A recent study has shown that hippocampal astrocytes indeed actively control the extracellular ion concentration (Wang et al., 2012a). In this study, it was found that G-protein mediated increases in astrocyte Ca^2+^ lead to an increase in uptake of K^+^ into astrocytes, which in turn leads to a slight neuronal hyperpolarization (Wang et al., 2012a). Similar results have been obtained in cerebellum (Wang et al., 2012b), suggesting that active regulation of the extracellular K^+^ concentration is a general mechanism by which astrocytes signal to neurons.

Another important function of astrocytes is the uptake of released neurotransmitters from the extracellular space. Through uptake, astrocytes limit the spread of neurotransmitters from the release site, thereby determining the spatiotemporal spread of transmitters (Tzingounis and Wadiche, 2007). Although it has been suggested that under control conditions astrocytes can efficiently clear synaptically released transmitters even upon high frequency stimulation (Diamond and Jahr, 2000), modulation of this uptake could results in changes in neuronal excitability and synaptic transmission. The efficacy of neurotransmitter clearance by astrocytes can be modulated either if astrocytes modulate their structural relationship with neurons, or if astrocytes change the amount or efficacy of their neurotransmitter transporters. Both mechanisms have been shown to occur under physiological conditions. An extreme example of a structural change occurs in the hypothalamus, where astrocytes dramatically retract their processes during lactation, parturition or chronic dehydration (for review see Theodosis et al., 2008). This retraction leads to an increase in glutamate spillover, and enables synaptically released glutamate to activate presynaptic mGluRs either on the same terminal (Oliet et al., 2001) or at neighboring synapses (Piet et al., 2004). Although the hypothalamus provides an extreme example of astrocytic reorganization, similar changes might occur in a more subtle way in other brain regions. For example, similar, albeit less dramatic, reorganization of astrocytic coverage has been shown to occur in the barrel cortex. Here, prolonged sensory activation leads to both a 2-fold increase in glial glutamate transporter levels, as well as an increased coverage of dendritic spines by astrocytic processes (Genoud et al., 2006). Through this mechanism active synapses would be equipped with a more efficient means of preventing crosstalk.

Thirdly, astrocytes can modulate neurons by releasing transmitters themselves. These so-called gliotransmitters are very diverse, including conventional transmitters like GABA and glutamate, as well as signaling molecules like purines, d-serine, taurine, cytokines, peptides, and metabolites like lactate (Volterra and Meldolesi, 2005). Astrocytes can release transmitters through two mechanisms. Firstly, they can release transmitter containing vesicles through SNARE mediated exocytosis. Astrocytes contain the necessary proteins for SNARE mediated exocytosis (Araque et al., 2000; Bezzi et al., 2004; Parpura and Zorec, 2010; Schubert et al., 2011), and genetic or pharmacological interference with proteins of the SNARE-complex in astrocytes inhibits numerous forms of astrocyte-neuron signaling (Pascual et al., 2005; Jourdain et al., 2007; Halassa et al., 2009; Henneberger et al., 2010; Min and Nevian, 2012). Secondly, transmitter can be released through reverse transport (Héja et al., 2009), or through membrane channels (Kozlov et al., 2006; Lee et al., 2010).

In conclusion, astrocytes can influence neurons by modulating the extracellular ionic composition, by changing their structural properties, or by releasing signaling molecules (gliotransmitters). But what is the impact of this on neuronal functioning? Numerous studies in different brain regions have uncovered a multitude of ways in which astrocytes can modulate neuronal excitability and synaptic transmission. In the following section we will review several of these different pathways.

## Astrocyte modulation of network excitability

The excitability of neurons is one of their most fundamental properties. Dynamic modulation of excitability is a powerful way of implementing state-dependent changes in neuronal computation. Several studies have shown that astrocytes can regulate neuronal excitability. Astrocytes can achieve this through several mechanisms: by regulation of the extracellular ionic composition, by maintaining a tonic extracellular transmitter concentration, by regulation of basal synaptic transmission, and by the induction of phasic events in neighboring neurons. We will shortly discuss these different mechanisms.

As described in the previous section, astrocytes can dynamically control the concentration of extracellular K^+^. Both hippocampal astrocytes (Wang et al., 2012a) as well as cerebellar Bermann glia translate an astrocytic Ca^2+^ signal into a transient decrease in the extracellular K^+^ concentration (Wang et al., 2012b). This decrease in extracellular K^+^ concentration leads to a transient hyperpolarization of adjacent neurons, thereby changing neuronal excitability. In hippocampus, this change in excitability is translated into a decrease in frequency and an increase in fidelity of excitatory synaptic transmission (Wang et al., 2012a). In cerebellum, paradoxically, the decrease in excitability is translated into an increase in the duration of Purkinje neuron upstates (Wang et al., 2012b). Therefore, astrocytes can dynamically modulate excitability through active modulation of extracellular ions.

Because astrocytes are responsible for the clearance of released neurotransmitters, they control the accumulation of these transmitters in the extracellular space. Therefore, astrocytes can regulate tonic neurotransmitter receptor mediated currents. For example, interfering with astrocyte uptake of GABA in the paraventricular nucleus leads to an increased tonic GABA(A) receptor mediated current, and a decrease in neuronal excitability (Park et al., 2009). Conversely, interfering with astrocytic glutamate uptake leads to increased excitability (Jabaudon et al., 1999; Pannasch et al., 2011). Interestingly, astrocytes can also regulate tonic transmitter concentration by releasing transmitter themselves. It was recently shown that tonic GABA mediated inhibition in the cerebellum is completely mediated by Bestrophin channel mediated GABA release from Bergman glia, and that interfering with these channels abolishes tonic inhibition (Lee et al., 2010). Furthermore, tonic GABA mediated inhibition in the hippocampus has been suggested to be mediated by reverse transport of GABA from astrocytes, and this mechanism was hypothesized to be boosted by astrocytic glutamate uptake (Héja et al., 2009). In this way, an increase in tonic inhibition would counterbalance increased excitatory neurotransmission. Therefore, astrocytes are in a prime position to control tonic inhibition and excitation of neurons, although it remains unclear whether this process is dynamically modulated.

In addition to controlling tonic currents, astrocytes are crucial for maintaining basal synaptic transmission. The astrocyte network provides energy to neurons in the form of lactate, and this energy supply is necessary to keep synaptic transmission intact (Rouach et al., 2008). Interestingly, the supply of energy is activity dependent, with lactate or its precursor glucose being able to travel through gap-junctions between astrocytes to reach the location where it is required (Rouach et al., 2008). In addition, astrocytes also set the level of basal synaptic strength. Two recent studies have shown that astrocytes are responsible for maintaining the basal release probability of excitatory synapses in the hippocampus at an elevated level (Di Castro et al., 2011; Panatier et al., 2011). In these studies, it was found that interfering with basal astrocyte signaling caused a reduction in synaptic efficacy in neighboring neurons in either the CA1 region (Panatier et al., 2011) or the dentate gyrus (Di Castro et al., 2011). The mechanism by which this regulation of basal transmission comes about seems to differ between the two brain regions. In CA1, astrocytes sense basal synaptic activity through mGluR5 activation, and maintain basal activity through activation of presynaptic adenosine A_2A_ receptors, which facilitate synaptic release (Panatier et al., 2011). In the dentate gyrus, astrocytes presumably sense synaptic corelease of ATP through P2Y1 receptors, and increase release through activation of presynaptic NMDA type glutamate receptors (NMDARs; Di Castro et al., 2011). However, it should be mentioned that two other studies have shown that selective stimulation of astrocytes through transgenic expression and activation of an exogenous receptor (Fiacco et al., 2007) or by genetically interfering with astrocyte Ca^2+^ signaling (Petravicz et al., 2008) has no effect on basal synaptic transmission in the CA1 region, thereby calling into question the importance of astrocytes for regulating basal synaptic transmission.

As a final mechanism by which astrocytes can regulate the excitable state of the network, astrocytes can physically release transmitters, leading to depolarization or hyperpolarization of neighboring neurons. Astrocytic glutamate release in the hippocampus can activate postsynaptic NMDARs, giving rise to so-called slow inward currents (SICs; Angulo et al., 2004; Fellin et al., 2004; Perea and Araque, 2005). SICs are NMDAR mediated slow events, typically lasting several hundreds of milliseconds. It is thought that one astrocytic glutamate release event can induce SICs simultaneously in several neighboring pyramidal neurons, which has led to the assumption that they play a role in the local synchronization of neurons (Angulo et al., 2004; Fellin et al., 2004). SICs have also been described in other brain regions, including the thalamus (Parri et al., 2001), spinal cord (Bardoni et al., 2010), and nucleus accumbens (D'Ascenzo et al., 2007). Interestingly, it was recently shown that, while activation of single astrocytes gives rise to SICs in neighboring neurons, simultaneous activation of a cluster (3–5) of astrocytes gives rise to a slower (>3 s) depolarization of neighboring neurons by 1–2 mV (Sasaki et al., 2011a). In contrast to SICs, this depolarization is mediated by non-NMDA glutamate receptors.

Analogous to the NMDAR mediated SICs that occur upon astrocytic glutamate release, astrocytic release of GABA can induce so-called slow outward currents (SOCs; Kozlov et al., 2006). These events have only been described in the olfactory bulb (Kozlov et al., 2006) and in the thalamus (Jiménez-González et al., 2011). Like SICs, SOCs occur simultaneously in neighboring neurons, so they might also play a role in neuronal synchronization. Interestingly, the source of GABA responsible for SOCs seems to be GABA flowing through a volume regulated anion channel, rather than vesicular GABA release (Kozlov et al., 2006).

Evidence for a role of astrocytically released neurotransmitters in the synchronization of neurons comes from experiments on slow cortical oscillations, which are formed by neuronal up- and down- states. Interfering with astrocyte signaling reduces the power of slow oscillations *in vivo* (Fellin et al., 2009), presumably through modulation of NMDARs and adenosine A1 receptors. Furthermore, *in vitro* experiments have shown that astrocytes control the frequency of cortical up-states (Poskanzer and Yuste, 2011).

In conclusion, astrocytes can modulate neuronal excitability by controlling the concentration of ions and neurotransmitters in the extracellular space, by regulating basal synaptic transmission, and by synchronously activating groups of neurons.

## Astrocytes and short-term synaptic plasticity

In addition to modulating neuronal excitability and basal synaptic transmission, astrocytes play a role in the specific strengthening or weakening of synaptic connections, either transiently (short-term plasticity), or long-lasting (long-term plasticity). Short-term plasticity (a change in synaptic strength lasting up to 10s of seconds) is thought to underlie critical computational functions of neuronal networks (Abbott and Regehr, 2004), whereas long-term plasticity (a change in synaptic strength lasting hours or longer) is involved in development, learning and memory formation. Short-term plasticity can be induced at three different levels: the conduction of the presynaptic action potential, the presynaptic release probability and the postsynaptic receptors. Strikingly, astrocytes have the ability to interfere with synaptic transmission at all these levels. Here we will review what is known about astrocyte involvement in short-term plasticity.

Recently, it was shown that glutamate release from astrocytes can activate axonal AMPARs (Sasaki et al., 2011b), and that this causes an axonal depolarization. The axonal depolarization leads to inactivation of axonal potassium channels, which causes a local broadening of the action potential waveform and an increase in presynaptic release probability (Alle and Geiger, 2006; Kole et al., 2007; Sasaki et al., 2011b). Through this mechanism, astrocytes can cause a transient increase in release probability in a population of neighboring synapses originating from the same axon.

As mentioned before, astrocytes can modulate basal synaptic transmission through tonic activation of presynaptic receptors (Di Castro et al., 2011; Panatier et al., 2011). However, if the activation of presynaptic receptors is transient, this leads to a short-term enhancement or depression of synaptic release probability. For example, phasic release of glutamatergic vesicles from astrocyte processes activates presynaptic glutamate receptors at several synapses. At excitatory CA3 to CA1 pyramidal neuron synapses in hippocampus, astrocyte activation by uncaging of Ca^2+^ or IP_3_ leads to astrocytic glutamate release, which in turn activates presynaptic mGluRs, leading to a transient increase in release probability (Fiacco and McCarthy, 2004; Perea and Araque, 2007). Importantly, this signaling cascade can also be activated by physiological astrocyte stimulation: a recent study has shown that activation of astrocytic CB_1_Rs by postsynaptically synthesized endocannabinoids leads to astrocytic glutamate release followed by mGluR mediated transient potentiation (Navarrete and Araque, 2010).

Astrocytic glutamate release can not only modulate synaptic transmission through activation of metabotropic receptors. There is also evidence that astrocytic glutamate release can activate presynaptically located ionotropic receptors. For example, in the hippocampal dentate gyrus, either electrical stimulation-induced or P2Y1 receptor mediated astrocyte activation leads to Ca^2+^ mediated exocytosis of glutamate, which activates presynaptic NMDARs (Jourdain et al., 2007; Santello et al., 2011). These receptors in turn induce a transient increase in release probability, as indicated by an increase in miniature excitatory postsynaptic current (mEPSC) frequency and an increased release probability at perforant-path synapses onto dentate granule cells (GCs). Interestingly, the astrocytic capacity to release glutamate is under tight regulation of tumor necrosis factor alpha (TNFα; Santello et al., 2011). In the absence of TNFα, altered vesicle docking dramatically slows down astrocytic glutamate release, leading to ineffective presynaptic NMDAR activation.

Another presynaptic ionotropic receptor that can be activated by astrocytic release of glutamate is the kainate receptor. At inhibitory synapses onto CA1 pyramidal neurons, presynaptic kainate receptors can increase release probability (Jiang et al., 2001). Ca^2+^ uncaging in astrocytes causes glutamate release which activates these presynaptic kainate receptors (Liu et al., 2004). More physiologically, repetitive interneuron firing activates this same pathway by activating astrocytic GABA(B) receptors through spillover of synaptically released GABA (Kang et al., 1998).

As mentioned before, astrocytes also release purines as gliotransmitters. Astrocyte derived ATP and its breakdown product adenosine mediate various forms of short-term synaptic plasticity. ATP released from astrocytes is quickly degraded to adenosine, and activation of presynaptic adenosine receptors can either lead to an increase or decrease in release probability, through activation of presynaptic A_2A_ and A_1_ receptors respectively. As mentioned before, astrocytic activation of presynaptic A_2A_ receptors maintains high basal release probability in the hippocampal CA1 region (Panatier et al., 2011). However, other studies have shown that astrocyte derived adenosine transiently decreases excitatory inputs to CA1 neurons through activation of presynaptic A_1_ receptors (Zhang et al., 2003; Pascual et al., 2005). The accumulation of astrocyte-derived adenosine during wakefulness (Schmitt et al., 2012) gradually increases this tonic presynaptic A_1_ receptor mediated suppression of synaptic transmission at these same synapses through activation of presynaptic A_1_ receptors, and this process is necessary for the buildup of sleep pressure (Halassa et al., 2009). Additionally, activity dependent release of astrocyte-derived purines mediates heterosynaptic short-term depression in CA1 neurons (Zhang et al., 2003; Pascual et al., 2005; Serrano et al., 2006). However, in this respect it is important to note that a recent publication disputes the glial origin of adenosine, arguing that heterosynaptic depression is not due to astrocyte derived ATP that is metabolized to adenosine, and instead suggests that adenosine is directly released from neurons (Lovatt et al., 2012).

In conclusion, several mechanisms have been described by which astrocytes can mediate short-term potentiation or depression. Interestingly, many of these processes have been studied at the same synapse: the excitatory Schaffer collateral synapse onto CA1 neurons of the hippocampus. From this, it can be concluded that Schaffer collateral synapses can either show short-term potentiation (Fiacco and McCarthy, 2004; Perea and Araque, 2007; Navarrete and Araque, 2010) or short-term depression (Zhang et al., 2003; Pascual et al., 2005; Serrano et al., 2006) upon astrocyte activation. Furthermore, it has also been suggested that astrocyte activation has no effect at all on synaptic transmission at Schaffer collateral synapses (Agulhon et al., 2010). Because the mentioned studies all differ in the way in which astrocytic activity was induced, a key question for future research is which physiological astrocyte stimuli lead to what kind of synaptic modulation. However, it is clear that astrocytes have a broad potential for modulating short-term synaptic plasticity.

## Astrocytes and long-term synaptic plasticity

Long-term synaptic plasticity is thought to be the cellular correlate of learning and memory (Bliss and Collingridge, 1993). Long-term plasticity can be divided into long-term potentiation (LTP), a long lasting increase in synaptic strength, and long-term depression (LTD), a long-lasting decrease in synaptic strength. LTP and LTD can be induced by a variety of protocols, including repetitive presynaptic stimulation (Bliss and Lomo, 1973; Kirkwood et al., 1993), precise timing of pre- and post-synaptic activity (spike-timing-dependent plasticity; STDP; Markram et al., 1997; Feldman, 2000; Sjöström et al., 2001) and application of pharmacological agents (Hosokawa et al., 1995; Lee et al., 1998). Furthermore, some forms of long-term plasticity can be homeostatic, meaning that the change in synaptic strength occurs at all synapses onto the neuron (Turrigiano and Nelson, 2004). Recent studies have shown that astrocytes are involved in many different forms of long-term plasticity. In the next section, we will review the literature on astrocyte involvement in long-term plasticity.

**Figure 2:**
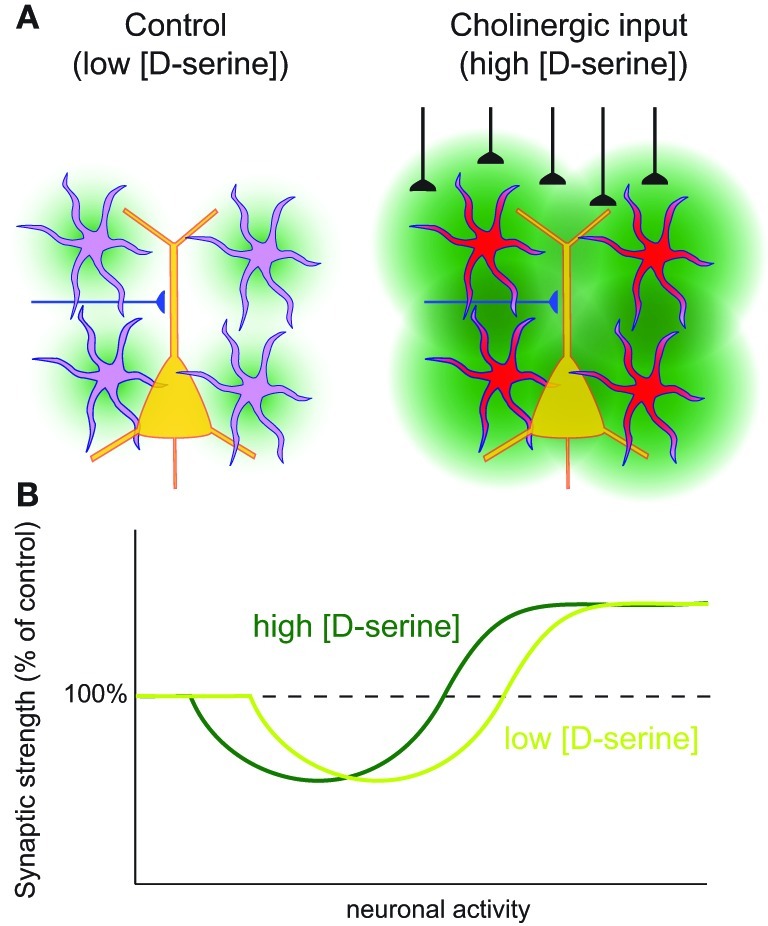
**Astrocyte mediated metaplasticity. (A)** Left: under basal conditions astrocytes release a basal tone of d-serine (green) which is sensed by neuronal NMDARs. Right: when astrocytes are stimulated, for example by the activation of cholinergic fibers, this leads to an increased release of d-serine from the astrocytes, which leads to an increased availability of neuronal NMDARs for activation. **(B)** Hypothetical BCM curve for the induction of LTP and LTD. Depending on the concentration of astrocyte-derived d-serine, the neuronal activity requirements for induction of LTP and LTD will shift.

Most forms of long-term synaptic plasticity depend on activation of postsynaptic NMDARs. The NMDAR classically is described as a coincidence detector of pre- and post synaptic neuronal activity. This is because for activation it needs both binding of presynaptically released glutamate as well as a postsynaptic depolarization to relieve it from block by magnesium (Mg^2+^) ions (Dingledine et al., 1999). Upon opening, NMDARs cause a synapse specific influx of Ca^2+^ into the postsynaptic neuron, which can activate second messenger cascades leading to either LTP or LTD (Malenka and Bear, 2004). However, a long ignored third requirement for NMDAR activation is the binding of a co-agonist to the receptor. NMDARs have a co-agonist binding site called the glycine binding site. The ion channel opens only when the glutamate binding site and the glycine binding site of the NMDAR are both occupied (Johnson and Ascher, 1987; Kleckner and Dingledine, 1988). The glycine binding site derives its name from the fact that glycine was first identified as an agonist at this site. However, recent reports have shown that d-serine is a more likely co-agonist for the NMDAR then glycine (Mothet et al., 2000). Interestingly, the source of d-serine in the brain seems to be the astrocyte. Astrocytes release d-serine into the extracellular space through vesicular fusion, and this astrocytic d-serine release is necessary to obtain sufficient NMDAR activation for induction of LTP in hippocampus (Yang et al., 2003; Henneberger et al., 2010) and prefrontal cortex (Fossat et al., 2011). Hippocampal LTD, like LTP, also requires activation of postsynaptic NMDARs. However, it has been suggested that while LTP requires high-frequency stimulation and therefore a strong activation of postsynaptic NMDARs, LTD comes about upon lower-frequency stimulation and a milder activation of postsynaptic NMDARs (Bienenstock et al., 1982; Artola and Singer, 1993; Figure 2B). Because LTD requires NMDAR activation, it also requires astrocytic d-serine release (Zhang et al., 2008).

A similar dependence of NMDAR mediated long-term plasticity on astrocyte-derived d-serine as shown in hippocampus was described in the hypothalamic supraoptic nucleus (SON; Panatier et al., 2006). In the SON, astrocytic coverage changes with behavioral state: during parturition and lactation astrocyte processes retract from the synapses. Under this condition the availability of astrocyte-derived d-serine for postsynaptic NMDARs is greatly reduced, leading to a shift in the activity dependence of LTP and LTD (Figure 2B).

Interestingly, a recent study showed that astrocytic release of d-serine can actively gate LTP in the rat barrel cortex *in vivo* (Takata et al., 2011). In this study, it was shown that stimulation of the nucleus basalis of Meynert, which provides the major cholinergic input to the barrel cortex, activates cortical astrocytes (Figure 2A). If this stimulation coincides with sensory stimulation, this leads to a potentiation of the sensory evoked potential. The authors show that this LTP of the sensory evoked potential depends on d-serine release from the astrocytes upon activation. These findings highlight the importance of d-serine as a modulator of plasticity both *in vitro* and *in vivo*. Furthermore, since cholinergic input into the cortex has been suggested to gate cortical plasticity (Bakin and Weinberger, 1996), these findings reveal astrocytes as a possible candidate mediating this gating.

Apart from acting as a NMDAR co-agonist, a recent study has shown that astrocytic release of d-serine can also induce synaptic plasticity independent of its role at the NMDAR (Kakegawa et al., 2011). In the immature cerebellum, burst stimulation of parallel fiber to Purkinje neuron synapses induced release of d-serine from Bergmann glia. This d-serine subsequently activated postsynaptic δ2 glutamate receptors, which in turn caused internalization of postsynaptic AMPARs. Interestingly, interfering with this form of LTD disrupted motor coordination *in vivo*, showing its relevance for cerebellar development. From the study by Kakegawa et al. (2011) it is not clear how burst stimulation of the parallel fiber synapses leads to activation of Bergmann glia. However, one exciting hypothesis that we would like to put forward is that endocannabinoids might mediate this process. Burst stimulation of parallel fiber synapses is known to cause the production and release of endocannabinoids from Purkinje neurons (Brenowitz and Regehr, 2005). Furthermore, LTD in the immature cerebellum requires endocannabinoid signaling (Safo and Regehr, 2005). Therefore, it is tempting to speculate that the activation of Bergmann glia by postsynaptically synthesized endocannabinoids could underlie the induction of LTD, which would be similar to recent findings on LTD in neocortex (Min and Nevian, 2012) and hippocampus (Han et al., 2012; see below). However, this hypothesis awaits further testing.

Another gliotransmitter that can mediate long-term plasticity is glutamate. Previously, we mentioned that astrocyte stimulation at hippocampal Schaffer collateral synapses can induce short-term potentiation through astrocytic glutamate release followed by activation of presynaptic mGluRs (Fiacco and McCarthy, 2004; Perea and Araque, 2007). Interestingly, this short-term potentiation can be transformed into LTP if the astrocyte activation is paired with a postsynaptic depolarization (Perea and Araque, 2007). It was recently demonstrated that this LTP mediated by coincident astrocytic and neuronal activity can be induced physiologically when excitatory transmission coincides with cholinergic input coming from the septal nucleus *in vivo* (Navarrete et al., 2012).

Additionally, a recent study showed that pharmacological activation of astrocytes *in vivo*, by application of the cannabinoid receptor agonist Δ^9^-THC, leads to LTD of Schaffer collateral synapses instead of LTP. This LTD also requires astrocytic glutamate release, but is mediated by activation of postsynaptic NMDARs, followed by endocytosis of postsynaptic AMPARs (Han et al., 2012). Therefore, depending on the type of stimulus, astrocyte activation can induce bidirectional hippocampal long-term plasticity.

**Figure 3:**
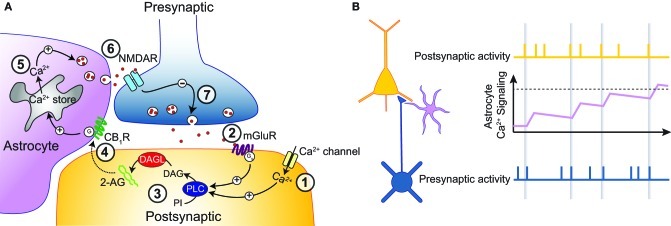
**Astrocytes as memory elements. (A)** Schematic representation of the pathway leading to t-LTD at developing neocortical synapses. A postsynaptic action potential leads to Ca^2+^ influx into the postsynaptic neuron (1). When this is followed by a presynaptic action potential, leading to activation of mGluRs (2), PLC is strongly activated (3), leading to the synthesis of endocannabinoids. These endocannabinoids leave the postsynaptic neuron to activate astrocytic CB_1_Rs (4), which cause release of Ca^2+^ from intracellular stores (5). The resultant Ca^2+^ transients trigger vesicular release of glutamate from the astrocyte, which in turn activates presynaptic NMDARs (6). This leads to a long-lasting depression in presynaptic release probability (7). **(B)** Astrocytes could act as memory elements. Schematic showing the action potential activity of a presynaptic (bottom, blue) and a postsynaptic (top, yellow) neuron, as well as the Ca^2+^ activity of a neighboring astrocyte (middle, pink). By sensing the buildup of endocannabinoids during subsequent pairings, astrocyte activity would gradually increase during the induction of t-LTD. t-LTD could then be induced only when astrocyte activity reaches a certain threshold (dotted line).

In the neocortex, we have recently shown that astrocytic release of glutamate is necessary for the induction of spike-timing-dependent depression (t-LTD; Min and Nevian, 2012), a form of long-term plasticity that is indispensable for sensory development (Li et al., 2009). In the developing sensory neocortex, excitatory synapses between L5 neurons and between L4 and L2/3 neurons contain presynaptic NMDARs (Sjöström et al., 2003; Bender et al., 2006; Nevian and Sakmann, 2006; Corlew et al., 2007; Rodríguez-Moreno and Paulsen, 2008). These receptors can modulate release probability directly (Sjöström et al., 2003; Brasier and Feldman, 2008), but additionally they are necessary for the induction of t-LTD of this synapse (Corlew et al., 2008). Until recently it was unclear whether these presynaptic NMDARs are autoreceptors for synaptically released glutamate, or if they are activated by glutamate coming from another source. Furthermore, while t-LTD was known to require activation of cannabinoid CB_1_Rs (Sjöström et al., 2003; Bender et al., 2006; Nevian and Sakmann, 2006), it was unclear how presynaptic NMDARs and CB_1_Rs together governed the induction of t-LTD. We showed that activation of presynaptic NMDARs during induction of t-LTD is mediated by astrocytic glutamate release (Min and Nevian, 2012; Figure 3A). Additionally, we found that postsynaptically synthesized endocannabinoids are the trigger for astrocyte activation during t-LTD induction. These endocannabinoids are only produced when a postsynaptic action potential is rapidly (within 10s of ms) followed by a presynaptic action potential. The reason for this narrow time window is the postsynaptic protein PLC, which acts as a coincidence detector (Hashimotodani et al., 2005) and is necessary for endocannabinoid synthesis (Nevian and Sakmann, 2006). Therefore, the precise timing window for the induction of t-LTD can be explained by the neuronal activity and coincidence detection upstream of the astrocyte activation. Subsequently, the synthesis of endocannabinoids leads to an increase in astrocytic Ca^2+^ signaling. This in turn leads to glutamate release from the astrocyte, which activates presynaptic NMDARs. Activation of the presynaptic NMDARs induces a long-lasting decrease in synaptic release probability, although the signaling cascade downstream from presynaptic NMDAR activation is still unclear (Figure 3A).

One interesting question rising from these results is how the presynaptic NMDARs are efficiently activated. Because the timing of the astrocytic Ca^2+^ signals, and therefore presumably the astrocytic glutamate release, is not correlated with respect to the pre- and post-synaptic action potentials (Min and Nevian, 2012), one would expect the Mg^2+^ block of the presynaptic NMDARs to hamper their efficient recruitment. However, it was recently shown that presynaptic NMDARs in the developing sensory cortex incorporate the NR3A subunit, which renders the receptor insensitive to Mg^2+^ block (Larsen et al., 2011). This modification would make presynaptic NMDARs ideal for sensing the temporally diffuse release of glutamate from astrocytes. However, it should also be noted that astrocyte stimulation by itself is not sufficient for the induction of t-LTD. Presynaptic action potential firing provides an additional requirement, although the mechanism behind this is currently unknown (Min and Nevian, 2012).

These results show that astrocytes form a crucial part of the retrograde signaling cascade for induction of t-LTD. Since endocannabinoid mediated forms of LTD occur in numerous brain regions and serve important functions (Heifets and Castillo, 2009), future studies will hopefully show whether astrocyte mediated retrograde signaling is a general principle for endocannabinoid mediated LTD.

Astrocytes also play a role in homeostatic control of synaptic transmission. This was first shown in cultured neurons, where glial release of the cytokine TNFα increases the synaptic expression of AMPARs, thereby controlling the weight of all excitatory synapses (Beattie et al., 2002). Another form of “multiplicative scaling” mediated by astrocytes was found in the hypothalamus. Here, exogenous stimulation of astrocytes by norepinephrine leads to astrocytic release of ATP, which subsequently activates postsynaptic P2X_7_ receptors. Activation of these postsynaptic receptors leads to insertion of AMPARs, and thereby to a long-lasting postsynaptic increase in the efficacy of excitatory synapses (Gordon et al., 2005). Interestingly, a more physiological protocol of repetitive stimulation of hypothalamic excitatory synapses can activate astrocyte Ca^2+^ signaling and subsequent release of ATP through mGluR activation. This protocol can lead to a similar long-lasting postsynaptic upscaling of the synaptic efficacy (Gordon et al., 2009). Therefore, astrocytes can play a role in homeostatic plasticity by translating global network activity into a distributed plasticity signal to multiple synapses.

Finally, long-term synaptic plasticity is costly in terms of energy. As mentioned before, astrocytic supply of energy to neurons is essential for maintaining synaptic transmission (Rouach et al., 2008). In addition, a recent study has shown that astrocytic energy supply is essential for the long-term maintenance of hippocampal LTP, and thereby for memory formation (Suzuki et al., 2011). In this study, it was found that learning was accompanied by an increase in the extracellular concentration of the astrocyte derived metabolite lactate. Blocking transport of lactate from astrocytes into the extracellular space, as well as blocking transport of lactate from the extracellular space into neurons interfered with the maintenance phase of LTP as well as with long-term memory (Suzuki et al., 2011). A similar involvement of lactate in memory formation was found in another recent study (Newman et al., 2011). These results show that not only receptor mediated astrocyte-neuron signaling but also metabolic support are crucial for long-term plasticity.

In conclusion, astrocytes play a role in several forms of long-term plasticity. Astrocytes can either set the threshold for LTP/LTD induction by controlling the extracellular concentration of d-serine, they can directly induce synaptic plasticity by releasing glutamate or other gliotransmitters, and they can mediate homeostatic plasticity. In addition, dynamic regulation of metabolic support by astrocytes is crucial for synaptic plasticity.

## The computational potential of astrocytes

In the previous section, we have discussed how astrocytes can modulate neuronal functioning. Based on the above described astrocyte-neuron interactions, we will summarize some possible roles that astrocytes could play in neuronal computation. We suggest that the functional role of astrocytes adds to the computational power of neuronal networks.

## Astrocyte mediated metaplasticity

Metaplasticity is a higher-order form of synaptic plasticity, defined as a change in the ability to induce synaptic plasticity (Abraham and Bear, 1996). There are several examples of neuronal activity patterns that do not induce synaptic plasticity, but that affect the threshold for subsequent induction of synaptic plasticity. Such metaplasticity has been proposed to play a role in several forms of learning (Abraham, 2008). Many forms of metaplasticity involve a change in the functionality of the postsynaptic NMDAR. Through the release of d-serine, astrocytes are ideally positioned to mediate metaplasticity. The level of astrocytic d-serine release will determine the possible amount of NMDAR activation and therefore of NMDAR mediated postsynaptic Ca^2+^ influx upon synaptic activity. Since many forms of LTP and LTD require NMDAR mediated Ca^2+^ influx for their induction, astrocytes can shift the threshold for LTP and LTD induction (Figure 2). In this way astrocytes can slide the threshold of the Bienenstock-Cooper-Munro (BCM) learning rule (Bienenstock et al., 1982; Artola and Singer, 1993). A clear example of this sliding threshold occurs in the hypothalamus where, as mentioned earlier, the amount of astrocytic coverage determines the synaptic concentration of d-serine and therefore, the induction threshold for LTP and LTD (Panatier et al., 2006). Furthermore, astrocytic d-serine release upon astrocyte activation by cholinergic inputs opens the window for subsequent LTP induction in the *in vivo* neocortex (Figure 2; Takata et al., 2011). Therefore, astrocytic d-serine release is a physiologically important metaplasticity signal in the brain. It can translate the activity state of the neuronal network into a modulatory signal that determines the corresponding learning rule: during a high activity state due to arousal or heightened attention the neuronal and astrocyte networks are highly active supporting the induction of LTP. Future behavioral studies should determine what the importance of this astrocyte mediated metaplasticity is for learning and memory formation.

## Astrocyte mediated heterosynaptic plasticity/synaptic clustering

Single astrocytes form non-overlapping domains (Bushong et al., 2002), and cover an area containing 300–600 dendrites, contacting up to 36 spines per dendrite (Halassa et al., 2007). This makes astrocytes well suited to signal information to a population of neighboring synapses. This means that astrocytes could be involved in forms of heterosynaptic plasticity: a plasticity signal generated at a single synaptic site could travel to neighboring synaptic sites to influence synaptic plasticity there. Furthermore, because astrocyte mediated heterosynaptic plasticity might not be confined to a single dendrite, the plasticity could affect synapses on neighboring dendrites even if they do not belong to the same neuron (so-called heteroneuronal plasticity). This requires that the locally induced increase in astrocytic Ca^2+^ signaling spreads along an astrocytic process and results in the release of gliotransmitters at neighboring synapses. Heterosynaptic short-term plasticity could play a role in switching between synaptic ensembles during information processing. As described earlier, astrocytes mediate heterosynaptic short-term depression at excitatory synapses onto CA1 pyramidal neurons (Zhang et al., 2003; Pascual et al., 2005; Serrano et al., 2006). Furthermore, endocannabinoid mediated astrocyte activation by stimulation of a single neuron induces heteroneuronal short-term potentiation at synapses onto non-stimulated neurons (Navarrete and Araque, 2010).

Heterosynaptic long-term plasticity has been implicated in the homeostatic control of synaptic inputs to a neuron (Chistiakova and Volgushev, 2009). Furthermore, heterosynaptic forms of long-term plasticity could facilitate the formation of clusters of neighboring synaptic inputs on dendrites (Larkum and Nevian, 2008). Such clusters have been proposed to be essential for the implementation of nonlinear synaptic integration in dendrites (Larkum et al., 2009; Legenstein and Maass, 2011). Several recent studies have shown the existence of clustered synaptic inputs (Kleindienst et al., 2011; Makino and Malinow, 2011; Takahashi et al., 2012). Whether astrocytes are able to fulfill a role in heterosynaptic long-term plasticity remains to be seen. However, it is interesting to note that several forms of endocannabinoid mediated LTD are heterosynaptic in nature (Chevaleyre and Castillo, 2003; Huang et al., 2008) and that astrocytes are implicated in some forms of endocannabinoid mediated LTD (Han et al., 2012; Min and Nevian, 2012).

It is important to note that not every activation of an astrocyte, manifested by increases in Ca^2+^ signaling, directly translates into synaptic plasticity. For example, we found that astrocyte activation with a voltage-clamp depolarization protocol results in increased Ca^2+^ signaling in the astrocyte, but that this activity alone does not change synaptic transmission strength at excitatory cortical synapses. An additional activation of the presynaptic axon was necessary in order to induce LTD (Min and Nevian, 2012). The astrocyte-stimulation-induced LTD (a-LTD) shares the same downstream signaling pathway with t-LTD, requiring activation of presynaptic NMDARs, but is independent of postsynaptic activity. This shows that additional factors have to be taken into account beyond astrocyte activation. A similar principle, where astrocyte activity is required but not sufficient for induction of plasticity, might explain the current controversy surrounding the involvement of astrocytes in hippocampal LTP (Agulhon et al., 2010; Henneberger et al., 2010; Smith, 2010). In this respect one further function that astrocytes might have in heterosynaptic modulation of synaptic transmission is that they lower the threshold for plasticity at neighboring synapses (heterosynaptic metaplasticity). In the case of a-LTD, release of glutamate onto presynaptic terminals that were previously not activated increases the propensity for LTD if these axons are activated within the time window of increased astrocyte activity. This can result in the above mentioned formation of clustered inputs onto neurons.

## Astrocytes as memory elements

The induction of many forms of synaptic plasticity requires a repeated presentation of a certain stimulus pattern (Petersen et al., 1998). An example of this is STDP, in which a single presentation of paired pre- and post-synaptic APs is insufficient for the induction of plasticity, but where repeated presentation of these stimuli over a period of several minutes can induce plasticity (O'Connor et al., 2005). This raises the question how a neuron can integrate transient coincident activity (on a millisecond timescale) over a period of minutes. Although it has been suggested that postsynaptic kinases or phosphatases could perform such integration (Lisman, 1989; Wang et al., 2005), astrocytes might also play an important role, since their basal timescale of signaling is several orders of magnitude slower than that of neurons. In support of this idea, we recently showed that the frequency of astrocytic Ca^2+^ transients gradually increases during the induction of t-LTD (Min and Nevian, 2012). This is achieved by the synthesis of endocannabinoids in the postsynaptic neuron during t-LTD induction. These endocannabinoids subsequently activate a neighboring astrocyte, and the astrocyte presumably senses the gradual buildup of the endocannabinoid concentration during repeated pairings (Figure 3A). The astrocyte then might act as a thresholding unit, releasing a retrograde t-LTD inducing signal onto the presynaptic neuron only when a sufficient level of astrocyte activation has been achieved (Figure 3B). In this way, astrocytes might act as memory elements for certain patterns of correlated neuronal activity.

How can an astrocyte act as a thresholding unit? The sophisticated Ca^2+^ signals in the processes of astrocytes can be used to perform chemical computations similar to linear and non-linear Ca^2+^ signaling in neurons. Efficient Ca^2+^ buffering (Neher, 1998), receptor clustering (Panatier et al., 2011; Arizono et al., 2012) and extrusion/uptake mechanisms could create Ca^2+^ microdomains that limit the release of gliotransmitters in space and time. Only certain patterns of Ca^2+^ signals might cause a high enough concentration of Ca^2+^ in the astrocyte processes to trigger vesicular release. This could be accomplished by local Ca^2+^ buffer saturation and a specific spatial relationship between Ca^2+^ release sites in the ER and the vesicular Ca^2+^ sensor triggering exocytosis (Marchaland et al., 2008; Bergersen et al., 2011). Therefore, a better understanding of the excitation secretion coupling and of microdomain Ca^2+^ signaling in astrocytes is needed to understand their chemical computation.

## How to link astrocyte mediated synaptic plasticity to behavior?

As described above, there is ample experimental evidence for an active role of astrocytes in neuronal plasticity. The most important question remaining is the functional relevance of the above described astrocyte-neuron interactions. This has proven very hard to address, since experimental approaches until recently were unable to specifically modify and study the role of astrocytes in neuronal computation and behavior *in vivo*. However, recent advances in genetics have made it possible to specifically alter astrocyte signaling *in vivo* while keeping neuronal signaling intact. This has led to some important insights into astrocyte involvement in information processing. For example, it is now clear that astrocyte derived adenosine modulates the build-up of sleep pressure as well as the cognitive effects of sleep deprivation (Halassa et al., 2009; Florian et al., 2011). Furthermore, using an astrocyte-specific knockout of the cannabinoid CB_1_ receptor, Han et al. (2012) showed that astrocytes are responsible for cannabinoid-induced reduction in working memory performance. Using astrocyte specific pharmacology, it was shown that lactate transport from astrocytes to neurons is crucial for long-term memory formation (Newman et al., 2011; Suzuki et al., 2011). Finally, a recent study showed that AMPARs found on Bergmann glia in the cerebellum are crucial for maintaining the integrity of glutamatergic synapses and that their deletion impairs fine motor coordination *in vivo* (Saab et al., 2012). Undoubtedly, future studies will shed more light on the role of astrocytes in behavior. It does however remain difficult to link behavioral readouts as are often obtained from *in vivo* experiments with the subcellular astrocyte-neuron signaling mechanisms as described in this review.

One possible way to bridge the gap between cellular mechanisms observed with high-resolution *in vitro* experiments and behavioral readouts *in vivo* is the use of mathematical models. As mentioned earlier, progress has been made with mathematical models of astrocyte Ca^2+^ signaling. In addition, several models have included bidirectional signaling between astrocytes and neurons on the synaptic level, leading to new insights into the function of this bidirectional signaling. For example, a study by Nadkarni et al. (2008) suggests that bidirectional signaling between astrocytes and presynaptic terminals can optimize synaptic information content by tuning presynaptic release probability (Nadkarni et al., 2008). In another study it has been suggested that astrocyte signaling can transiently switch the direction of short-term synaptic plasticity between paired-pulse depression and facilitation (De Pittà et al., 2011). Such models are a starting point for the development of realistic neuron-glia networks.

An example of a recently developed neuron-glia network model comes from a recent study by Wade et al. (2011). Here, it was shown that astrocyte mediated SICs can synchronize pre- and post-synaptic neuronal responses. When combining this with a neuron-dependent STDP rule, the authors show that astrocyte mediated synchronization could act as a “learning signal” at remote synaptic sites (Wade et al., 2011). Another recent study shows the learning potential of neuron-glia networks (Porto-Pazos et al., 2011). In this study, the inclusion of astrocytes into a multi-layer feed forward neuronal network model designed to solve different classification tasks improved performance. Interestingly, increased performance seemed to come at the expense of learning speed. Astrocytes in this model were responding to highly active neuronal connections, and in turn bidirectionally regulated synaptic weights with a slow temporal time-course. The increase in performance could not be explained by the addition of extra elements to the neuronal network, since keeping the number of elements constant led to a similar increase in performance. However, it crucially depended on the response properties of the added astrocytes, with the astrocytes integrating neuronal activity over longer time-scales as well as being active for longer times then the neurons (Porto-Pazos et al., 2011). It should be noted that the astrocyte-neuron network implemented in this study has a simplified structure: each synapse in the network was controlled by a single-independent astrocyte. Therefore, the spread of activity through the astrocyte network, which might lead to heterosynaptic or homeostatic plasticity, was not included. Future work implementing more realistic astrocyte-neuron networks capturing features described in this review might lead to novel insights into how astrocytes contribute to neuronal computation. Based on the improvement seen with rudimentary astrocyte-neuron networks, we predict that network performance will be greatly enhanced when realistic astrocyte-neuron signaling is implemented.

In conclusion, research on the involvement of astrocytes in neuronal signaling in the last years has resulted in a rich array of possible astrocyte-neuron interactions. It is now evident that beyond their permissive role in synaptic and network function astrocytes can perform integration of neuronal signals by means of their Ca^2+^ dynamics, thereby potentially enhancing the computational power of neuronal networks. The challenge for the future is to understand how and when astrocyte mediated signaling processes are involved in computation in the brain. To answer this fascinating question will require a combination of state-of-the-art experimental techniques with advanced computational modeling.

### Conflict of interest statement

The authors declare that the research was conducted in the absence of any commercial or financial relationships that could be construed as a potential conflict of interest.
